# Basic Symptoms Are Associated With Age in Patients With a Clinical High-Risk State for Psychosis: Results From the PRONIA Study

**DOI:** 10.3389/fpsyt.2020.552175

**Published:** 2020-11-17

**Authors:** Helene Walger, Linda A. Antonucci, Alessandro Pigoni, Rachel Upthegrove, Raimo K. R. Salokangas, Rebekka Lencer, Katharine Chisholm, Anita Riecher-Rössler, Theresa Haidl, Eva Meisenzahl, Marlene Rosen, Stephan Ruhrmann, Joseph Kambeitz, Lana Kambeitz-Ilankovic, Peter Falkai, Anne Ruef, Jarmo Hietala, Christos Pantelis, Stephen J. Wood, Paolo Brambilla, Alessandro Bertolino, Stefan Borgwardt, Nikolaos Koutsouleris, Frauke Schultze-Lutter

**Affiliations:** ^1^Department of Psychiatry and Psychotherapy, Ludwig-Maximilian-University, Munich, Germany; ^2^Department of Education, Psychology, Communication, University of Bari Aldo Moro, Bari, Italy; ^3^Department of Neurosciences and Mental Health, Istituto Di Ricovero e Cura a Carattere Scientifico (IRCCS) Ca' Granda Foundation Major Hospital Polyclinic, University of Milan, Milan, Italy; ^4^MoMiLab Research Unit, Institutions, Markets, Technologies (IMT) School for Advanced Studies Lucca, Lucca, Italy; ^5^Institute for Mental Health, University of Birmingham, Birmingham, United Kingdom; ^6^Department of Psychiatry, Medical Faculty, University of Turku, Turku, Finland; ^7^Department of Psychiatry and Psychotherapy, and Otto Creutzfeldt Center for Cognitive and Behavioral Neuroscience, University of Muenster, Muenster, Germany; ^8^Department of Psychology, Aston University, Birmingham, United Kingdom; ^9^Department of Psychiatry (Psychiatric University Hospital, UPK), University of Basel, Basel, Switzerland; ^10^Department of Psychiatry and Psychotherapy, Faculty of Medicine and University Hospital Cologne, University of Cologne, Cologne, Germany; ^11^Department of Psychiatry and Psychotherapy, Medical Faculty, Heinrich-Heine University, Düsseldorf, Germany; ^12^Melbourne Neuropsychiatry Centre, Department of Psychiatry, The University of Melbourne and Melbourne Health, Carlton, VIC, Australia; ^13^Orygen, The National Centre of Excellence for Youth Mental Health, Melbourne, VIC, Australia; ^14^Centre for Youth Mental Health, University of Melbourne, Parkville, VIC, Australia; ^15^Group of Psychiatric Neuroscience, Department of Basic Medical Science, Neuroscience, and Sense Organs, University of Bari Aldo Moro, Bari, Italy; ^16^Department of Psychology and Mental Health, Faculty of Psychology, Airlangga University, Surabaya, Indonesia

**Keywords:** psychosis, clinical high risk, basic symptoms, age, brain maturation

## Abstract

In community studies, both attenuated psychotic symptoms (APS) and basic symptoms (BS) were more frequent but less clinically relevant in children and adolescents compared to adults. In doing so, they displayed differential age thresholds that were around age 16 for APS, around age 18 for perceptive BS, and within the early twenties for cognitive BS. Only the age effect has previously been studied and replicated in clinical samples for APS. Thus, we examined the reported age effect on and age thresholds of 14 criteria-relevant BS in a patient sample at clinical-high risk of psychosis (*N* = 261, age 15–40 yrs.), recruited within the European multicenter PRONIA-study. BS and the BS criteria, “Cognitive Disturbances” (COGDIS) and “Cognitive-perceptive BS” (COPER), were assessed with the “Schizophrenia Proneness Instrument, Adult version” (SPI-A). Using logistic regressions, prevalence rates of perceptive and cognitive BS, and of COGDIS and COPER, as well as the impact of social and role functioning on the association between age and BS were studied in three age groups (15–18 years, 19–23 years, 24–40 years). Most patients (91.2%) reported any BS, 55.9% any perceptive and 87.4% any cognitive BS. Furthermore, 56.3% met COGDIS and 80.5% COPER. Not exhibiting the reported differential age thresholds, both perceptive and cognitive BS, and, at trend level only, COPER were less prevalent in the oldest age group (24–40 years); COGDIS was most frequent in the youngest group (15–18 years). Functional deficits did not better explain the association with age, particularly in perceptive BS and cognitive BS meeting the frequency requirement of BS criteria. Our findings broadly confirmed an age threshold in BS and, thus, the earlier assumed link between presence of BS and brain maturation processes. Yet, age thresholds of perceptive and cognitive BS did not differ. This lack of differential age thresholds might be due to more pronounced the brain abnormalities in this clinical sample compared to earlier community samples. These might have also shown in more frequently occurring and persistent BS that, however, also resulted from a sampling toward these, i.e., toward COGDIS. Future studies should address the neurobiological basis of CHR criteria in relation to age.

## Introduction

Despite their low lifetime prevalence of between 0.2 and 3.5% (1), schizophrenia-spectrum and other psychotic disorders are among the most severe and costly neuropsychiatric diseases (2–4). Schizophrenia is the 9th most important cause for disability-adjusted life-years (DALYs) already in 10–14-year-old boys, and the 2nd most important in all 15–19-year-olds (5), although full-blown psychoses rarely manifest in children and adolescents (6). However, because the majority of psychoses develop slowly over several years, their first prodromal symptoms will frequently show in childhood and adolescence; and prodromes with such an early onset tend to be longer and to be associated with poorer outcome (7, 8). Thus, the early detection and prevention of psychosis, which aims to reduce the burden of the disorder (9–11), has increasingly moved from adult patients into ever younger patient groups. However, this has been done without full consideration of potentially influential developmental issues (6, 12, 13).

In the early detection of psychoses, two complementary approaches to define the clinical high-risk (CHR) state for psychosis are currently followed (9, 14). One is the ultra-high-risk (UHR) approach that was developed to detect an imminent risk of psychosis. It includes (1) the attenuated psychotic symptoms (APS) syndrome characterized by recently developed or worsened symptoms that resemble positive symptoms of psychosis, yet with still some insight being maintained; (2) the brief limited intermittent psychotic symptoms (BLIPS) syndrome with frank positive psychotic symptoms that spontaneously remit within a couple of days; and (3) the genetic risk plus functional decline (GRFD) syndrome that combines a significant recent functional decline with a genetic risk factor of psychosis, i.e., a first-degree relative with psychosis or a schizotypal personality disorder in the patient (9, 14).

The second approach is the basic symptoms (BS) approach that was developed to detect emerging psychosis as early as possible (9, 14). It includes two alternative criteria, “Cognitive disturbances” (COGDIS) and “Cognitive-perceptive BS” (COPER) (Table 1). BS have been described as the earliest subtle and subjectively experienced symptoms of psychosis (16–19). BS are subclinical disturbances in the affected individual's own mental processes, such as thinking, speech, (body) perception, motor action, drive, affect, and stress tolerance, that are primarily recognized by the affected individual and are only rarely directly observable by others (16–19). BS were named “basic” as they had been assumed to be “substrate-close,” i.e., the most immediate psychopathological expression of the neurobiological aberrations underlying the development of psychotic disorders. At this, BS are assumed to be the basis on which (attenuated) psychotic symptoms develop as a result of dysfunctional coping (16, 18). By definition, BS are experienced with immediate and full insight and, thus, are distinct from APS, BLIPS and frank psychotic symptoms, which, at least initially, are experienced as being real and/or reasonable (10, 16, 17, 19).

**Table 1:** Basic Symptom (BS) criteria.

Cognitive disturbances (COGDIS)
Presence of ≥ 2 of the following 9 basic symptoms of at least moderate severity (≥3)^a^ within the last 3 months
Inability to divide attention
Thought interference
Thought pressure
Thought blockages
Disturbance of receptive speech
Disturbance of expressive speech
Unstable ideas of reference
Disturbance of abstract thinking
Captivation of attention by details of the visual field
Cognitive-perceptive basic symptoms (COPER)
Presence of ≥ 1 of the following 10 basic symptoms of at least moderate severity (≥3)^a^ within the last 3 month and first occurrence ≥12 months ago
Thought interference
Thought perseveration
Thought pressure
Thought blockages
Disturbance of receptive speech
Decreased ability to discriminate between ideas/perception, fantasy/true memories
Unstable ideas of reference
Derealization
Visual perception disturbances *(excl. blurred vision and hypersensitivity to light)*
Acoustic perception disturbances *(excl. hypersensitivity to sound/noises)*

a *Assessed by the Schizophrenia Proneness Instrument (SPI-A) (15)*.

As a result of the above, models of the early course of psychosis commonly assume that BS and BS criteria develop first, and are followed by UHR symptoms and criteria before more persistent psychotic symptoms set in (9, 10, 14, 16, 17). This sequence, however, was only partially supported by retrospective analyses of first-episode psychosis patients (7, 20). However, the temporal sequence ‘BS-APS-positive symptoms' was particularly frequent in first-episode psychosis patients with an onset of first prodromal symptoms before age 18 (7).

UHR and BS criteria have been associated with pooled long-term conversion rates into full-blown psychosis between 37 and 61%, with higher conversion rates in BS-defined samples compared to UHR samples at observation periods of three or more years, and with significantly lower conversion rates in child and adolescent compared to adult samples (14). Next to this lower psychosis-predictive value of CHR criteria in children and younger adolescents, in particular of the APS syndrome (14, 21), accumulating evidence suggests that age and developmental aspects might also alter the general clinical relevance and the prevalence rate of CHR symptoms. Again, this evidence has mainly accumulated for APS and BLIPS (13, 14, 21–28), in doing so indicating an age threshold around the age of 16 below which these symptoms are more frequent, but less clinically relevant in terms of their association with functional deficits and/or non-psychotic mental disorder, and less psychosis-predictive (21–23, 28, 29). In doing so, the interaction of APS with age played a particular role in predicting psychosocial functioning, with APS being increasingly associated with functional deficits with advancing age in the community (29). The only exception was disorganized communication at APS-level for that, in interaction with age, no stable association with functional deficits was revealed. Non-psychotic mental disorders, however, were mainly predicted independently by female sex and presence of APS (29). Again, disorganized communication was an exception in that it predicted mental disorder by its interaction with age rather than its sole presence. In doing so, participants without disorganized communication were commonly younger than those with it; this age effect being more pronounced in those with mental disorder (29).

As regards BS and BS criteria, the impact of age and developmental aspects has been less studied. Two studies on the dimensions of BS indicated differences between children and adolescents, and adult patients (19, 30). Yet, only one Swiss community study has so far studied age effects on the prevalence and clinical relevance of BS (29, 31). It reported age thresholds of around 18 years of age for perceptive BS and of within the first half of the twenties for cognitive BS, indicating a higher prevalence and a lesser clinical relevance, i.e., a lesser association with functional impairment, below these age thresholds (29, 31). As in APS, age played a major role in moderating the association between BS and functional deficits, while female sex was an independent predictor of mental disorder (29). In doing so, the association of BS and psychosocial functioning increased with age. Only in case of perceptual BS, their interaction with age was additionally moderated by sex, indicating that the effect of the interaction between age and perceptual BS on psychosocial impairment was more pronounced in males (29). In case of non-psychotic mental disorders, in addition to the general sex effect, age only moderated the effect of cognitive BS, indicating that a mental disorder was less likely when participants with cognitive BS were younger (29). Thus, in both APS and BS, age commonly moderated their association with functional deficits but not their association with mental disorder (29).

**Figure 1:**
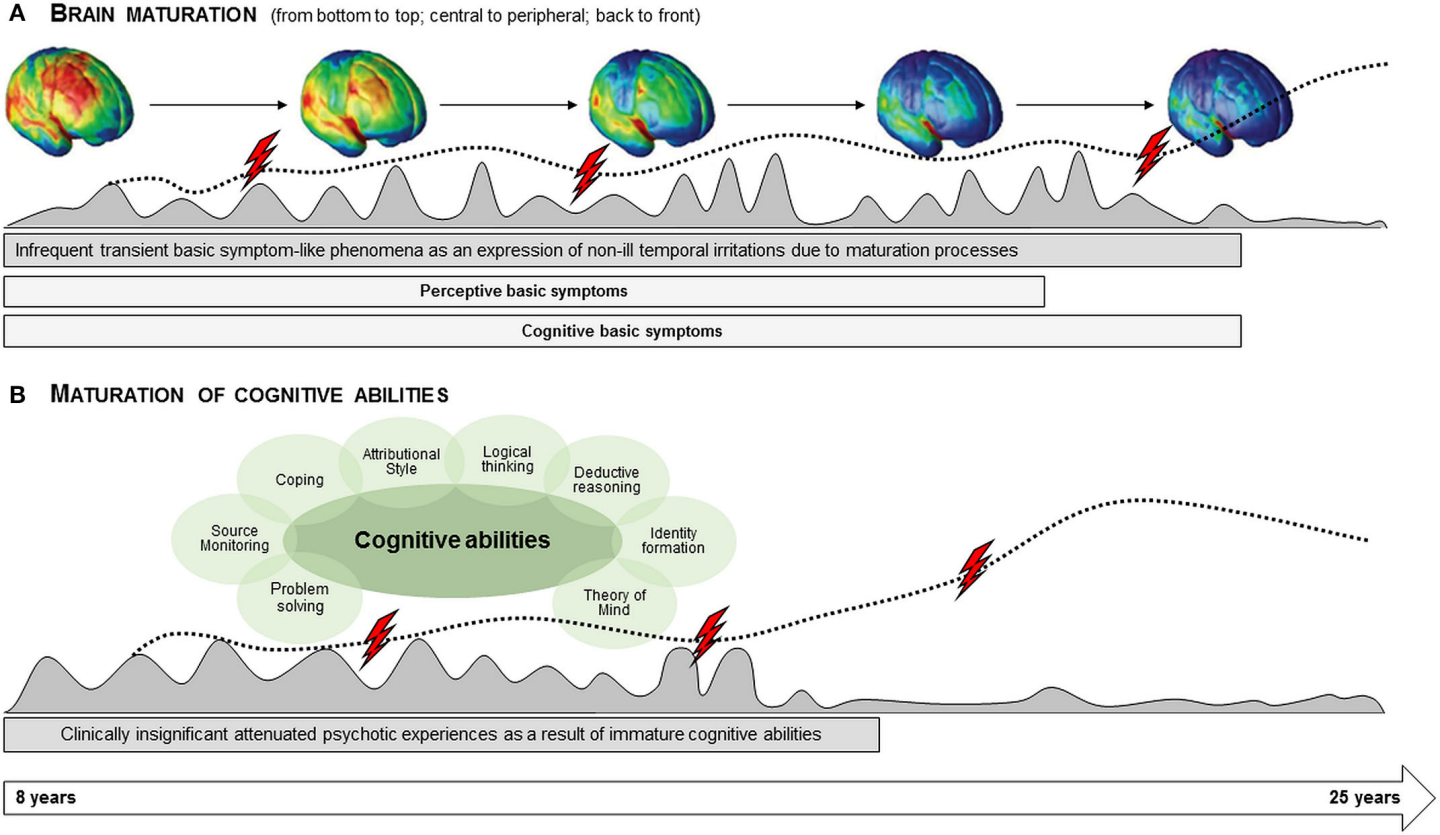
Model of the possible relationship between basic symptoms and brain maturation, and between attenuated psychotic symptoms and maturation of cognitive abilities (31). This model assumes that **(A)** subtle subclinical disturbances in cognitive and perceptive processes that are phenomenologically identical to basic symptoms (BS) might occur during childhood and adolescence as infrequent temporary expressions of insignificant passing dysfunctions in the wake of brain maturation processes (gray-shaded curve). However, if these disturbances, i.e., BS, occur more frequently (i.e., meet the frequency requirements of the BS criteria) and are persistent (dotted line), they might be a sign of disturbances in brain maturation processes, which, in line with a neurodevelopmental model of psychosis (32, 33), might predispose to the development of psychosis. A genetic predisposition, childhood adversities or other risk factors (indicated by flashes) including stressful life-events and cognitions promoting the development of attenuated psychotic symptoms (APS), such as poor coping, externalization biases or poor source monitoring (34), might further sustain and/or amplify the development and persistence of information processing disturbances. On the other hand, the model assumes that **(B)** unusual perceptual experiences or thought contents phenomenologically identical to APS might occur during childhood and early adolescence as an expression of not yet fully matured cognitive abilities (gray-shaded curve). If their maturation is impaired by risk factors or stressors (flashes) or neurodevelopmental disturbances in information processing (that might be experienced as BS), APS might persist or progress (dotted line), potentially leading to positive and potentially benign schizotypal traits (35), an Attenuated Psychosis Syndrome (36), and/or psychosis.

In light of current models and the assumed sequence of BS before UHR symptoms in the at-risk or prodromal stage of psychosis (9, 10, 14, 16, 17), the higher age thresholds of BS (31) compared to APS (22) in the Swiss community study were surprising, because a lower or at least similar age threshold of BS compared to APS had been expected (31). Yet, the two age thresholds of perceptive and cognitive BS seemed to follow known brain maturation processes (29, 31). Consequently, the higher prevalence of BS below these thresholds in concert with the fact that BS mostly occurred too infrequently to meet the BS criteria's frequency requirement of “at least once per week” (Table 1) was explained by ongoing brain maturation processes (31). According to the suggested model (Figure 1), the prevalent yet rarely occurring BS below the respective age thresholds were proposed to signal subtle, transient disruptions of mental processes that occur as part of the ongoing transformations at the cerebral level, and thus to support the proposed substrate-closeness of BS (31). The age threshold of APS, however, seemed to reflect the age at which most cognitive abilities have been acquired (31, 37). As a result, APS that were more prevalent below this threshold and also mainly occurred more rarely than the required frequency of “at least once per week” (22) were explained as the expression of not yet fully matured cognitive abilities, including coping strategies, which makes the young person more prone to develop inadequate explanatory models (Figure 1) (31).

In light of these considerations that may have major impact on future research into the neurobiological underpinnings of symptoms of psychosis (15, 31) and the future development of both age-adapted early detection and age-adapted early interventions (6, 12, 31), and in light of the limited studies on age effects on BS, we investigated the age effects on and age thresholds of BS in a CHR sample. In line with the replication of age effects on APS in clinical samples (23, 25), we expected that age thresholds would follow those earlier reported from the Swiss community study (29, 31). Additionally, in light of the moderating effect of age on the association of BS with functional impairment, which was weaker in younger subjects (29, 31), we also examined if the effect of age on BS might be influenced by the presence of functional impairments.

## Methods

### Sample

The sample consisted of 261 patients with a CHR state who were recruited as part of the EU-funded Personalized Prognostic Tools for Early Psychosis Management (PRONIA) study [www.pronia.eu (38)] at ten early-detection centers in five European countries between February 2014 and November 2017 (Table 2). In each center, the study was approved by the local ethics committee, and all participants or participant's parents/guardians gave their written informed consent prior to study inclusion.

**Table 2:** Sociodemographic characteristics and prevalence of at least one of the 14 basic symptom (BS), irrespective of BS criteria's frequency and novelty requirements.

**Characteristic**	**Subjects with** **≥** **1 of 14 BS (*****n*** **=** **238)**		**Subjects without any of 14 BS (*****n*** **=** **23)**		**Total sample (*****n*** **=** **261)**		**Statistics^a^**			
	**Mdn**	**Mean (SD)**	**Mdn**	**Mean (SD)**	**Mdn**	**Mean (SD)**	***U***		***p***	**ES**
Age	21.5	23.0 (± 5.3)	26.0	25.0 (± 6.4)	22.0	23.3 (± 5.5)	1,934.5	0.020		0.144
Educational years	13.0	13.5 (± 2.7)	14.0	13.9 (± 3.1)	13.0	13.5 (± 2.7)	1,299.5	0.253		0.076
	***N***	***%***	***N***	***%***	***N***	***%***	***x***^**2**^	***df***	***p***	**ES**
Age group (n)							5.087	2	0.079	0.140
15–18 yrs.	40	95.2^b^	2	4.8^b^	42	16.1^b^				
19–23 yrs.	110	94.0^b^	7	6.0^b^	117	44.8^b^				
24-40 yrs.	88	86.3^b^	14	13.7^b^	102	39.1^b^				
Center							8.280	9	0.506	0.178
LMU Munich	83	34.9	11	47.8	94	36.0	1.527	1	0.217	0.076
UBS Basel	23	9.7	0	0.0	23	8.8	2.437	1	0.118	0.097
UKK Cologne	34	14.3	3	13.0	37	14.2	0.027	1	0.870	0.010
University Birmingham	16	6.7	2	8.7	18	6.9	0.127	1	0.721	0.022
University Turku	27	11.3	0	0.0	27	10.3	2.910	1	0.088	0.106
University Udine	17	7.1	3	13.0	20	7.7	1.032	1	0.310	0.063
University Milan	20	8.4	2	8.7	22	8.4	0.002	1	0.962	0.003
University Münster	11	4.6	2	8.7	13	5.0	0.735	1	0.391	0.053
University Bari	1	0.4	0	0.0	1	0.4	0.097	1	0.755	0.019
University Düsseldorf	6	2.5	0	0.0	6	2.3	0.593	1	0.441	0.048
Gender: male	114	47.9	10	43.5	124	47.5	0.164	1	0.685	0.025
Migratory background^c^	28	11.8	2	8.7	30	11.5	0.194	1	0.659	0.027
Any current non-psychotic axis-I disorder	151	63.4	8	34.8	159	60.9	7.237	1	0.007	0.167
Any major depressive disorder	117	49.2	5	21.7	122	46.7	6.335	1	0.012	0.156
1st-degree biological relative with psychosis	36	15.1	1	4.3	37	14.2	2.003	1	0.157	0.088
Schizotypal personality disorder	17	7.1	2	8.7	19	7.3	0.075	1	0.784	0.017
Any UHR-criterion	172	72.8	15	65.2	187	71.6	0.513	1	0.474	0.044
BLIPS syndrome	5	2.1	0	0.0	5	1.9	0.493	1	0.483	0.043
APS syndrome	154	64.7	13	56.5	167	64.0	0.610	1	0.435	0.048
GRFD syndrome	36	15.1	3	13.0	39	14.9	0.072	1	0.789	0.017
COGDIS	147	61.8	0	0.0	147	56.3	32.523	1	0.001	0.353
COPER	210	88.2	0	0.0	210	80.5	103.858	1	0.001	0.631
impaired GF *social*^d^ ≤ 6	122	51.3	8	34.8	130	49.8	2.278	1	0.131	0.093
impaired GF *role*^e^ ≤ 6	130	54.6	13	56.5	143	54.8	0.031	1	0.861	0.011

a *Test statistics: Mann-Whitney U-test for non-normally distributed continuous variables with Rosenthal's r as effect size (ES), chi-squared test for categorical variables with Cramer's V as effect size (ES)*.

b *% of age group not of BS group sample*.

c *any other white/Asian/black/mixed background*.

d GF social = Global Functioning social scale a value of ≤ 6 indicates presence of “moderate impairment in social/interpersonal functioning.”

e GF role = Global Functioning (GF) role scale a value of ≤ 6 indicates presence of “moderate impairment of role functioning.”

The inclusion criteria of the CHR sample were age between 15 and 40 years, sufficient knowledge of the local language, in which the assessments were conducted, and meeting any one of the slightly adapted UHR criteria (Supplementary Table 1) and/or COGDIS (Table 1). Participants were excluded in case of a past or present diagnosis of psychosis and of treatment with any antipsychotic medication at or above the minimum dosage threshold defined by the DGPPN S3 Guidelines for the treatment of first-episode psychosis (2006) (39) for either more than 30 days or within the past 3 months before baseline assessment (Supplementary Table 2). Further exclusion criteria were an IQ below 70, alcohol or polysubstance dependence within the past 6 months, current or past head trauma with loss of consciousness for more than 5 min, or any neurological or somatic disorder having a potential effect on the brain.

### Assessments

For the assessment of the UHR criteria, the Structured Interview for Psychosis-Risk Syndromes (SIPS) (40) was used. The BS criteria COPER and COGDIS were assessed with the Schizophrenia Proneness Instrument – Adult version (SPI-A) (41). Following the procedures of the Swiss community study (22, 23, 31), cognitive and perceptive BS were distinguished: Cognitive BS comprised at least any one of the following 12 BS: inability to divide attention; thought interference, pressure, blockages and perseveration; disturbances of receptive and expressive speech, of abstract thinking, or of discriminating between ideas and perceptions; unstable ideas of reference; capturing of attention by details of the visual field and derealization. Perceptive BS included at least any one of the various visual or acoustic perception disturbances. Additionally, BS were distinguished according to meeting or not meeting the novelty requirement (i.e., BS constitutes a disruption in the person's “normal” self and has a distinct time of first occurrence) and the frequency requirement (i.e., BS occurs at least once per week within the past 3 months) of the BS criteria.

Furthermore, the Structured Clinical Interview for DSM-IV-TR (SCID) (42) was performed to rule out past or present psychosis and to assess other mental disorders. Moreover, the Global Functioning: Social (GF: S) (43) and Global Functioning: Role (GF: R) (43) as well as the Global Assessment of Functioning (GAF) (44) were used to measure level of psychosocial functioning both globally and specifically. Impaired psychosocial functioning was assumed when the global GAF score was 70 or lower and when a GF score was 6 or lower.

All interviewers were trained in the assessments. Additionally, weekly supervision within each research center and monthly CHR case conferences on the CHR criteria relevant for inclusion by phone with an expert in early detection of psychoses (F.S.-L.) were carried out to ensure excellent and reliable data quality across centers.

### Statistical Analyses

Using SPSS 25.0, frequencies were compared by chi-squared test for categorical variables, and non-normally distributed interval and ordinal data were analyzed with the Mann-Whitney U test.

As in previous studies of age effects (22, 23, 29, 31), binary logistic regression analysis with “enter” as method were used to evaluate effects of defined age ranges on prevalence rates of “at least any one of the 14 BS,” “at least any one cognitive BS” and “at least any one perceptive BS” as well as of the related novelty and frequency requirements in the total CHR sample (*N* = 261). Because our age range differed from earlier studies (22, 23, 29, 31) by not including the age range of 8–14 years, we aligned the definition of our age groups with the results on BS in the community (29, 31) and defined three age groups: 15–18 years for the age threshold around age 18 reported for perceptive BS, 19–23 years because of the age threshold of within the first half of the twenties reported for cognitive BS, and 24–40 years. Also roughly in line with the previous studies (22, 23, 31), the age group of 19–23 years was used as the reference group in regression analyses. This age group was chosen, because the peak onset of first-episode psychosis was reported to be between the ages of 20 and 24 years (45) and, thus, this age group can be expected to show the highest rate of CHR symptoms and criteria. The reliability of the regression results was internally examined using bootstrapping (*N* = 1,000).

Furthermore, stepwise logistic regression analyses (Wald method) were employed to test effects of age and of functional deficits in both social and role functioning as well as of their interaction on the respective presence of BS and the BS criteria requirements. More specifically, “age group,” “GF: S ≤ 6” or “GF: R ≤ 6” and “age group” × “GF: S ≤ 6”/”GF: R ≤ 6” were entered as potential predictors and the respective BS variable entered as dependent variable. To ensure stable results, the effects were only considered as significant when the same predictors were selected in both forward and backward selection.

## Results

Presence of at least one of the 14 BS within 3 months prior to the interview was reported by 238 patients (91.2%) (Table 2). Out of these, 87.4 % reported cognitive BS and 55.9 % reported perceptive BS. Furthermore, the COGDIS criterion was met by 147 (56.3%) and the COPER criterion by 210 patients (80.5%); naturally, all of them were members of the group with BS (Table 2). Any UHR criterion was reported by 187 participants (71.6%), mainly by meeting the APS syndrome (64.0%). There was no difference in frequency of UHR criteria between those with and without BS. Patients with BS were on average 2 years younger than those without any BS and more frequently presented with any non-psychotic axis-I disorder, in particular major depressive disorder (Table 2). No significant difference was seen between those with and without BS with regard to educational years, recruitment site, sex, migration background, family history of psychosis, presence of schizotypal personality disorder or of functional impairment (Table 2).

Logistic regression analyses indicated a lower frequency for both perceptive and cognitive BS, and the related requirements, in those of age 24 and over compared to the two younger groups, i.e., the 15–18-year-olds and the reference group of 19–23-year-olds (Table 3). Overall, this age effect was slightly more pronounced for perceptive BS compared to cognitive BS, with Odds Ratios [i.e., Exp (ß)] being between 0.073 and 0.004 points lower. For all 14 BS together, this difference only reached the level of a statistical trend for overall prevalence and the frequency requirement (Table 3).

**Table 3:** Effects of age on the report of the 14 BS irrespective of novelty and frequency (a), when meeting novelty requirement (b) and when meeting frequency requirement (c); binary logistic analysis with method “enter” and 19–23-year-olds as the reference age group.

**Age group (years)**	**ß**	**SE**	**Wald (*df* = 1)**	***p* after bootstrap**	**Exp (ß)**	**95% CI**	***n* present**	**% present**
**(A) PREVALENCE OF BS IRRESPECTIVE OF NOVELTY AND FREQUENCY**								
**≥** **1 BS (in 19–23 years:** ***n*** **=** **110, 94%)**								
15–18	0.241	0.823	0.086	0.769	1.273	0.25–6.38	40	95.2
24–40	***−0.916***	***0.485***	***3.577***	***0.059***	***0.400***	***0.16–1.03***	88	86.3
**≥** **1 cognitive BS (in 19–23 years:** ***n*** **=** **107, 91.5%)**								
15–18	−0.119	0.621	0.037	0.848	0.888	0.26–2.99	38	90.5
24–40	**−0.896**	**0.417**	**4.612**	**0.032**	**0.408**	**0.18–0.93**	83	81.4
**≥** **1 perceptive BS (in 19–23 years:** ***n*** **=** **77, 65.8%)**								
15–18	0.147	0.387	0.145	0.703	1.159	0.54–2.47	29	69.0
24–40	**−1.093**	**0.281**	**15.105**	**<** **0.001**	**0.335**	**0.19–0.58**	40	39.2
**(B) PREVALENCE OF BS** ***MEETING NOVELTY*** **AND IRRESPECTIVE OF FREQUENCY REQUIREMENT**								
**≥** **1 BS (in 19–23 years:** ***n*** **=** **109, 93.2%)**								
15–18	0.384	0.812	0.223	0.636	1.468	0.3–7.20	40	95.2
24–40	**−0.930**	**0.456**	**4.153**	**0.042**	**0.394**	**0.16–0.97**	86	84.3
**≥** **1 cognitive BS (in 19–23 years:** ***n*** **=** **104, 88.9%)**								
15–18	−0.078	0.56	0.019	0.889	0.925	0.31–2.78	37	88.1
24–40	–**0.845**	**0.378**	**5.010**	**0.025**	**0.429**	**0.21–0.90**	79	77.5
**≥** **1 perceptive BS (in 19–23 years:** ***n*** **=** **75, 64.1%)**								
15–18	0.223	0.385	0.333	0.564	1.249	0.59–2.66	29	69.0
24–40	–**1.018**	**0.28**	**13.242**	**<** **0.001**	**0.361**	**0.21–0.63**	40	39.2
**(C) PREVALENCE of BS** ***MEETING FREQUENCY*** **AND IRRESPECTIVE OF NOVELTY REQUIREMENT**								
**≥** **1 BS (in 19–23 years:** ***n*** **=** **101, 86.3%)**								
15–18	0.409	0.591	0.479	0.489	1.505	0.47–4.79	38	90.5
24–40	–***0.609***	***0.359***	***2.881***	***0.090***	***0.544***	***0.27–1.10***	79	77.5
**≥** **1 cognitive BS (in 19–23 years:** ***n*** **=** **97, 82.9%)**								
15–18	0.423	0.536	0.621	0.431	1.526	0.53–4,36	37	88.1
24–40	–**0.656**	**0.329**	**3.964**	**0.046**	**0.519**	**0.27–0.99**	73	71.6
**≥** **1 perceptive BS (in 19–23 years:** ***n*** **=** **55, 47%)**								
15–18	0.120	0.360	0.111	0.739	1.127	0.56–2.28	21	50.0
24–40	–**0.663**	**0.283**	**5.504**	**0.019**	**0.515**	**0.30–0.90**	32	31.4

*Significant predictors (p < 0.05) are in bold type, predictors with significance at statistical trend (p < 0.10) in bold italics*.

As regards BS criteria, COPER revealed a statistical trend toward being least frequent in the oldest age group (Table 4). COGDIS was significantly more frequent in the youngest age group, i.e., in 15–18-year-olds (Table 4).

**Table 4:** Effects of age on the report of BS criteria COPER and COGDIS; binary logistic analysis with method “enter” and 19–23-year-olds as the reference age group.

**Age group (years)**	**ß**	**SE**	**Wald (*df* = 1)**	***p* after bootstrap**	**Exp (ß)**	**95% CI**	***N present***	**% present**
**Cognitive-perceptive BS (COPER; in 19–23 years:** ***n*** **=** **98, 93.8%)**								
15–18	0.151	0.507	0.089	0.766	1.163	0.43-3.14	36	85.7
24–40	–***0.568***	***0.338***	***2.818***	***0.093***	***0.567***	***0.29–1.1***	76	74.5
**Cognitive disturbances (COGDIS; in 19–23 years:** ***n*** **=** **60, 51.3%)**								
15–18	**0.751**	**0.382**	**3.874**	**0.049**	**2.119**	**1.0–4.48**	**29**	**69.0**
24–40	0.225	0.272	0.682	0.409	1.252	0.73–2.14	58	56.9

*Significant predictors (p < 0.05) are in bold type, predictors with significance at statistical trend (p < 0.10) in bold italics*.

Regarding the potential influence of the functional level on the presence of BS or BS criteria, the results indicated that only age rather than impaired social or role functioning or their interaction with age was a predictor of the presence of BS and BS criteria (Tables 5, 6). Yet, compared to univariate analyses of age effects, age was less frequently selected as a significant predictor in the multivariate analyses (Tables 5, 6).

**Table 5:** Age effects on BS considering impaired global social functioning (GF social ≤ 6).

	**Significant predictor**	**ß**	**SE**	**Wald (*df* = 1)**	***p* after bootstrap**	**Exp (ß)**	**95% CI**
***Prevalence of BS irrespective of novelty and frequency requirements***							
≥ 1 BS	No significant predictor						
≥ 1 cognitive BS	No significant predictor						
≥ 1 perceptive BS	**Age**	–**0.684**	**0.191**	**12.838**	**<** **0.001**	**0.505**	**0.35–0.73**
***Prevalence of BS meeting novelty irrespective of frequency requirement***							
≥ 1 BS	* Age*	**–*****0.637***	***0.334***	***3.633***	***0.057***	***0.529***	***0.28–1.02***
≥ 1 cognitive BS	No significant predictor						
≥ 1 perceptive BS	**Age**	–**0.664**	**0.190**	**12.262**	**<** **0.001**	**0.515**	**0.36–0.75**
***Prevalence of BS meeting frequency irrespective of novelty requirement***							
≥ 1 BS	No significant predictor						
≥ 1 cognitive BS	**Age**	–**0.571**	**0.241**	**5.628**	**0.018**	**0.565**	**0.35–0.91**
≥ 1 perceptive BS	**Age**	–**0.403**	**0.182**	**4.908**	**0.027**	**0.668**	**0.47–0.96**
***Prevalence of BS criteria***							
COPER	No significant predictor						
COGDIS	No significant predictor						

*Binary logistic analysis with method “forward” and “backward” using the respective BS as dependent variable; Age group, GF social ≤ 6 and “age group x GF social ≤ 6” entered as predictors. Only stable models are reported (both methods revealed significant effects). Significant predictors (p < 0.05) are in bold type, predictors with significance at statistical trend (p < 0.10) in bold italics*.

**Table 6:** Age effects on BS considering impaired global role functioning (GF role ≤ 6).

	**Significant predictor**	**ß**	**SE**	**Wald (*df* = 1)**	***p* after bootstrap**	**Exp (ß)**	**95% CI**
***Prevalence of BS irrespective of novelty and frequency requirements***							
≥ 1 BS	No significant predictor						
≥ 1 cognitive BS	No significant predictor						
≥ 1 perceptive BS	**Age**	–**0.684**	**0.191**	**12.838**	**<** **0.001**	**0.505**	**0.35–0.73**
***Prevalence of BS meeting novelty irrespective of frequency requirement***							
≥ 1 BS	*Age*	**–*****0.668***	***0.340***	***3.853***	***0.050***	***0.513***	***0.26–1.0***
≥ 1 cognitive BS	No significant predictor						
≥ 1 perceptive BS	**Age**	–**0.664**	**0.190**	**12.262**	**<** **0.001**	**0.515**	**0.36–0.75**
***Prevalence of BS meeting frequency irrespective of novelty requirement***							
≥ 1 BS	No significant predictor						
≥ 1 cognitive BS	**Age**	–**0.571**	**0.241**	**5.628**	**0.018**	**0.565**	**0.35–0.91**
≥ 1 perceptive BS	**Age**	–**0.248**	**0.107**	**5.389**	**0.020**	**0.780**	**0.63–0.97**
***Prevalence of BS criteria***							
COPER	No significant predictor						
COGDIS	No significant predictor						

*Binary logistic analysis with method “forward” and “backward” using the respective BS as dependent variable; age group, GF role ≤ 6 and “age group x GF role ≤ 6” entered as predictors. Only stable models are reported (both methods revealed significant effects). Significant predictors (p < 0.05) are in bold type, predictors with significance at statistical trend (p < 0.10) in bold italics*.

## Discussion

This is the first study to examine the effect of age and developmental aspects in a CHR sample, alternatively defined by UHR criteria and COGDIS. Within our clinical CHR sample, 91.2% reported the presence of any of the 14 BS included in the definition of COGDIS and COPER. Expectedly and largely independent of functional deficits, our analyses showed a significant lower prevalence of BS in the older age group, i.e., in patients of and above age 24, with the relative probability of reporting BS decreasing by roughly 35–40%.

The previously described age thresholds for perceptive BS around late adolescence (i.e., around age 18) and for cognitive BS in the early twenties (31) that seemed to follow brain maturation processes were considered as further support of the assumption that BS were the most immediate psychopathological expressions of neurobiological aberrations underlying the disorder, i.e., that they were “substrate-close” (29, 31). Hence, the higher prevalence of usually infrequently occurring BS in younger age groups in the community was regarded as indicating that low-frequency BS might occur in childhood and adolescence as an infrequent and temporary non-pathological expression of insignificant and transient dysfunctions in the wake of normal brain maturation (31). Thus, given an undisturbed, “normal” brain maturation, these non-pathological BS would spontaneously remit – or grow out again – over the course of further maturation processes (31) (Figure 1). In the Swiss community sample (31), however, BS had not only been less frequent than in our sample (18.1 vs. 91.2%), they had also been reported as meeting frequency requirements of BS criteria, i.e., as occurring at least once per week, at a far lower rate (in only 33.7 vs. 90.7% of those with BS). Whereas, the rate of those meeting the novelty requirement had been only slightly lower in the community (in 77.8 vs. 98.7% of those with BS). A higher frequency of BS along with a higher persistence, which had not been examined in both the community and the present study, has been assumed to indicate disturbances in brain maturation that might predispose to the development of psychosis (31) (Figure 1). Consequently, our present results from a more mentally unwell clinical sample suggest that they might not exhibit the same differential age thresholds for perceptive and cognitive BS for the very reason that the frequent occurrence of BS already signals aberrant neurodevelopment. If this was true, BS should also be more persistent in clinical samples; thus, the course of BS needs to be examined in future studies. For this reason, future psychopathological and neurobiological studies should not only assess the age-of-onset of BS but also examine differences between subjects with an onset of BS before and those with an onset after the likely conclusion of major brain maturation processes in the early twenties.

Furthermore, from the community results it was assumed that an onset before the early twenties may reflect aberrant brain maturation, while an onset at an older age may reflect neurodegenerative processes (31). The possibility to distinguish between aberrant neurodevelopmental and neurodegenerative processes might well-impact on the choice of treatment, for example between neuroprotective and anti-inflammatory interventions (46–48). Thus, a simple reliable and valid marker to guide this distinction, such as age-at-onset and course of BS, might greatly enhance the development of benign psychopharmacotherapy in CHR states.

Next to the clinical status, sampling differences might have also caused the missing differential age thresholds, in particular the selection bias in favor of cognitive BS that meet novelty and frequency criteria, the more restricted age range of the PRONIA study with no inclusion of 8–14-year-olds, the low number of patients below age 19 (16.1%), and the dominance of the reference age group of 19–23-year-olds (44.8%). These biases resulted in low numbers of cases without any BS and without any cognitive BS and, relatedly, higher standard errors (SE; Table 3) in the youngest group, which increase the confidence intervals and the probability (*p*) to falsely accept the null hypothesis of equality between the youngest and the reference group. Thus, from a statistical point of view, the oldest age group that had a more favorable ratio between positive and negative cases, offered a higher likelihood to detect differences between age groups. Compared to cognitive BS, a higher likelihood to detect differences between age groups was also given for perceptive BS that are not part of COGDIS (Table 1) and that, consequently, were not directly influenced by the inclusion criteria. Because COPER and COGDIS tend to frequently co-occur because of their shared cognitive BS (49, 50), and our results might reflect a slightly more “natural” and robust variance in relation to age in perceptive BS compared to cognitive BS that was thus maintained in the multivariate models including functional deficits. In contrast, in the community sample, the perceptive BS had shown less pronounced age effects as compared to cognitive BS (31). In light of these differences in sampling and clinical status between the earlier community (29, 31) and our clinical sample, the missing difference between the age thresholds does not seem surprising. Rather, overall, our findings seem to support the notion that criteria-relevant BS occur more frequent before the conclusion of brain maturation in the first half of the twenties. The assumed close link of BS to the “substrate,” of course, requires validation in future neurobiological studies, for example in structural imaging studies for that a decrease in gray matter volume (GMV) would be expected to be related to BS groups as a result of potential aberrant brain maturation leading, for example, to an excess in pruning (18, 51, 52) or to neurodegenerative, for example, inflammatory processes (53, 54). In respect to the model on both BS and UHR symptoms (Figure 1), however, an increase in GMV would be expected to be related to UHR symptoms as a result of excessive compensatory – though inadequate – neurocognitive coping processes in response to other symptoms or environmental stressors (34, 55).

Thus, the combined assessment of BS and symptomatic UHR criteria might help to better understand brain imaging results in UHR samples reporting both GMV decrease (in the right gyrus rectus, the right superior frontal gyrus, and the left superior frontal gyrus) and GMV increase (in the bilateral median cingulate, the right fusiform gyrus, the left superior temporal gyrus, and the right thalamus) (56). The GMV-increased primary auditory and neocortical language regions, superior temporal gyrus, and insula were reported as the core regions responsible for the positive symptoms, such as delusions, hallucinations, and disorganized speech (57–59) and, in longitudinal studies, progressive GMV reduction of the superior temporal gyrus was linked to low improvement in positive psychotic symptoms (60, 61). These GMV increases are still subject to debate and discussed in relation to different pathophysiological processes in the early phase, age, other demographic differences, genetic predisposal, and different MRI scanners or parameters employed in the method section (56). In light of the model in Figure 1, however, the increase in regions already related specifically to positive symptoms might well be perceived as a result of excessive neurocognitive and psychological processes, i.e., intensive cognitive activities, that might have also become evident by the increase in thalamic structures responsible for the emotional experience and expression, and cognitive functions, such as memory, attention, and sensory-guided actions (56). On the other hand, all three regions with GMV reductions are involved in cognitive processes that might be reflected by criteria-relevant cognitive BS (Table 1). These processes are: (1), working memory (62), possibly impaired in thought blockages; (2) complex attention (63), possibly impaired in inability to divide attention and captivation of attention, (3) response inhibition (64), possibly impaired in thought interference, pressure and perseveration, disturbance of abstract thinking and unstable ideas of reference; and (4) language and memory recall (65), possibly impaired in disturbances of receptive and expressive speech, and disturbance in distinguish between memory and phantasy. These possible relations between APS and BS and GMV aberrations should be studied in future imaging studies using fine-grained psychopathological measures (15).

### Strengths and Limitations

Our study has several strengths and limitations. Among the strengths are clearly the large sample size and the high-quality assessment of BS. Next to the discussed limitations related to sampling, a clear limitation is the related impossibility to reanalyze the age effect on APS for lack of patients below the suggested age threshold of 16 years. Yet, this age threshold has already been replicated in clinical samples (23) and, thus, can be assumed to work in the present sample. Furthermore, the fact that the age thresholds in BS reported from the Swiss community sample (29, 31) could be replicated in this clinical sample, despite the differences in age range, suggest that these findings may generalize to other samples.

### Outlook

As the early detection of psychosis is increasingly applied to ever younger age groups, the need to re-evaluate the validity and clinical significance of current CHR criteria and symptoms in younger age groups should be addressed in future studies to improve understanding of what properties (such as age-at-onset, frequency and persistence) convey their clinical relevance at different developmental levels. In doing so, their association with objective measures, such as imaging-based tools, should be given additional attention to gain further insight into the pathogenesis of psychosis and its early symptoms. Such studies have the potential to gain insights into useful targets for interventions and, thus, to improve outcomes prior to the first manifestation of psychosis.

## Data Availability Statement

The raw data supporting the conclusions of this article will be made available by the authors, without undue reservation.

## Ethics Statement

The studies involving human participants were reviewed and approved by local research ethics committees of each location. Written informed consent to participate in this study was provided by the participants' legal guardian/next of kin.

## Author Contributions

HW wrote the first draft under supervision of FS-L and they had full access to the data and take responsibility for the integrity of the data and the accuracy of the data analysis. HW, NK, LK-I, SR, MR, AR, KC, JK, TH, FS-L, AR-R, RU, JH, CP, SW, PB, and SB: acquisition, analysis, or interpretation of data. HW, FS-L, LA, and AP: statistical analysis. NK, LK-I, SR, AR-R, RS, CP, PB, SB, and SW: obtained funding. NK, MR, AR, KC, TH, RU, JH, EM, PB, and SB: administrative, technical, or material support. FS-L, NK, LA, and AP: supervision. All other authors critically revised the manuscript for important intellectual contents and approved of the final version of the manuscript.

## Conflict of Interest

NK received honoraria for talks presented at education meetings organized by Otsuka/Lundbeck. CP participated in advisory boards for Janssen-Cilag, AstraZeneca, Lundbeck, and Servier and received honoraria for talks presented at educational meetings organized by AstraZeneca, Janssen-Cilag, Eli Lilly, Pfizer, Lundbeck, and Shire. RU has received honoraria for talks presented at educational meetings organized by Sunovion. The remaining authors declare that the research was conducted in the absence of any commercial or financial relationships that could be construed as a potential conflict of interest.
