# Supplementary material for: Basic Symptoms Are Associated With Age in Patients With a Clinical High-Risk State for Psychosis: Results From the PRONIA Study
Source: Front Psychiatry. 2020 Nov 17;11:552175. doi: 10.3389/fpsyt.2020.552175 (PMC7707000; doi:10.3389/fpsyt.2020.552175)
Supplement: Supplementary file 1 [file Data_Sheet_1.docx]

**Online supplementary materials**

**to**

**Basic symptoms are associated with age in patients with a clinical high-risk state for psychosis: results from the PRONIA study**

**by H. Walger et al.**

**eTable 1** Adapted PRONIA ultra-high risk (UHR) criteria^a^

| **A. Presence of at least any 1 of 5 attenuated psychotic symptom (APS)** with a moderate to severe, but not psychotic, severity (SIPS score 3-5)^b^ that (1) began within the past year or was rated one or more scale points higher compared to 12 month ago, and (2) occurred at an average frequency of at least once per week for at least several minutes per event in the past month | |
| --- | --- |
| Unusual thought content/delusional ideas |  |
| Suspiciousness/persecutory ideas |  |
| Grandiosity |  |
| Perceptual abnormalities/hallucinations |  |
| Disorganized communication |  |
| **B. Presence of brief limited intermittent psychotic symptoms (BLIPS)** defined by one of the symptoms listed above (1) reaching a psychotic level of intensity in each of the past 3 months for at least several minutes per day, OR (2) reaching a psychotic level of intensity in the past month, occurring at an average frequency of at least once per week for at least several minutes per event in the past month, or occurring at least for a cumulative period of more than one hour within the past month, AND (1+2) remitting spontaneously within one week (i.e. without antipsychotic medication) | |
| **C. Genetic risk and functional deterioration (GRFD)** state, defined by a current 30% or greater reduction in the functional disability score of the split version of the Global Assessment of Functioning Scale (GAF) [1] compared with the highest lifetime level of functioning, and having a first-degree relative with a history of any psychotic disorder, or having a DSM-IV-TR schizotypal personality disorder^c^ | |

^a^ Presence of at least one of A, B or C to meet PRONIA clinical high-risk (CHR) criteria

^b^ Assessed by the Structured Interview for Psychosis-Risk Syndromes (SIPS) [2]

^c^ Assessed by the Structured Clinical Interview for DSM-IV-TR (SCID) [3]

**References to eTable 1**

1. Pedersen, G., K.A. Hagtvet, and S. Karterud, Generalizability studies of the Global Assessment of Functioning-Split version. Compr Psychiatry, 2007. 48(1): p. 88-94 DOI: 10.1016/j.comppsych.2006.03.008.

2. McGlashan, T., B. Walsh, and S. Wood, The psychosis risk syndrome: Handbook for diagnosis and follow-up 2010, Oxford University Press, New York.

3. First MB, W.J. BW, S. RL, and G. M, Structured Clinical Interview DSM-IV-TR Axis I Disorders, Research Version, Patient Edition. (SCID-I/P). 2002, New York: Biometrics Research, New York State Psychiatric Institute.

| Substance | Recommended starting dosage  (mg/d) | Dosage interval | Target dosage first-episode psychosis  (mg/d) | Target dosage relapsing schizophrenia (mg/d) | Maximum dosage recommended  (mg/d) |
| --- | --- | --- | --- | --- | --- |
| Atypical Antipsychotics | | | | | |
| Amisulpride | 200 | (1)-2 | 100-300 | 400-800 | 1200 |
| Aripiprazole | (10)-15 | 1 | 15-(30) | 15-30 | 30 |
| Olanzapine | 5-10 | 1 | 5-15 | 5-20 | 20 |
| Quetiapine | 50 | 2 | 300-600 | 400-750 | 750 |
| Risperidone | 2 | 1-2 | 1-4 | 3-6-(10) | 16 |
| Ziprasidone | 40 | 2 | 40-80 | 80-160 | 160 |
| Typical Antipsychotics | | | | | |
| Fluphenazine | 0.4-10 | 2-3 | 2.4-10 | 10-20 | 20-(40) |
| Flupentixole | 2-10 | 1-3 | 2-10 | 10-60 | 60 |
| Haloperidole | 1-10 | (1)-2 | 1-4 | 3-15 | 100 |
| Perazine | 50-150 | 1-2 | 100-300 | 200-600 | 1000 |
| Perphenazine | 4-24 | 1-3 | 6-36 | 12-42 | 56 |
| Pimozide | 1-4 | 2 | 1-4 | 2-12 | 16 |
| Zotepine | 25-50 | 2-(4) | 50-150 | 75-150 | 450 |
| Zuclophentixole | 2-50 | 1-3 | 2-10 | 25-50 | 75 |

**eTable 2** DGPPN S3 Guidelines for the treatment of first-episode psychosis and schizophrenia (2006) (translated English version of Table 4.1 stated in the short version of the guideline manual available in <https://www.dgppn.de/_Resources/Persistent/a6e04aa47e146de9e159fd2ca1e6987853a055d7/S3_Schizo_Kurzversion.pdf>)

Participants were excluded in case of treatment with any antipsychotic medication at or above the minimum dosage threshold defined by the DGPPN S3 Guidelines for the treatment of first-episode psychosis for either more than 30 days or within the past three months before baseline assessment.
